# Hand skeletal maturity and its correlation with mandibular dental development

**DOI:** 10.4317/jced.51433

**Published:** 2014-07-01

**Authors:** Ali Bagherpour, Maryam Pousti, Elahe Adelianfar

**Affiliations:** 1 Associate Professor of Oral and Maxillofacial Radiology, Dental Research Center, School of Dentistry, Mashhad University of Medical Sciences, Mashhad, Iran; 2 Assistant Professor, Orthodontic Department, Dental Branch, Islamic Azad University, Tehran, Iran; 3 Private Dental Practice, Mashhad, Iran

## Abstract

Objectives: Assessment of pubertal status and pubertal growth spurt in orthodontic patients has a considerable impact on the diagnosis, treatment plan and the outcome of orthodontic treatments. Hand-wrist radiography is routinely used to evaluate skeletal development. Some studies have shown that there is an association between bone development and different stages of dental calcification; therefore, the stages of dental calcification can be used as the first tool for diagnosis, in case there is an association. This study was performed with the aim to evaluate the association between the phases of dental development and the stages of skeletal maturity. Study design: In this study, a total of 52 patients (26 males and 26 females), referring to Mashhad School of Dentistry for orthodontic treatment, were evaluated; the subjects were within the age range of 9-14 years. Hand-wrist radiographic evaluation of skeletal maturation was performed using Fishman method. Also, the Demirjian method was used to identify the stages of dental calcification by panoramic radiographs. Independent t-test and Spearman correlation coefficient were used for data analyses. Results: The mean age of males and females was 11.05 ± 1.05 and 10.62 ± 1.12 years, respectively (*p*=0.156). The Spearman correlation coefficients between skeletal maturity indicators (SMIs) and developmental stages of mandibular left and right canines and second molars were significant in males only (*p*<0.05). Also, correlation coefficients were significant between adductor sesamoid ossification and mandibular right and left canines developmental stages in males (*p*<0.05). Conclusions: Findings of this study showed that the correlation between dental developmental stages and skeletal maturity only were significant in males; thus, different skeletal maturity patterns in males and females might be perceptible.

** Key words:**Skeletal maturation, hand-wrist radiography, panoramic radiography.

## Introduction

Assessment of pubertal status and pubertal growth spurt has a considerable impact on the diagnosis, objectives, design, and the results of orthodontic treatments. This influence is most evident when a significant increase or decrease of craniofacial growth affects various treatments including: the application and time of using extra-oral tractions, use of functional appliances, therapy with or without tooth extractions, selecting and applying different retention types in orthodontics, and time of orthognathic surgery (1-6).

In orthodontic treatments, the assessment of the patient in terms of the remaining growth potential has a great significance (7); it is important to check if the patient is reaching the peak growth, is in the peak, or has passed it. This is the theory behind the correlation between the diagnosable stages of the skeletal development of hand and wrist and pubertal growth. Many indicators are cited in various articles, among which the calcification of adductor sesamoid bones of the thumb is considered as an indicator of peak height velocity [PHV] increase (8,9).

Unfortunately, little information exists regarding the relationship between skeletal maturation and stages of dental development. Dental development, especially in the case of mandibular second molar and canine tooth, and its association with skeletal maturation have been studied. This study was performed with the aim to evaluate the association between the developmental stages of mandibular second molars and canines teeth in panoramic radiographs with the indicators of puberty growth spurt in hand-wrist radiography; the subjects were adolescents within the age range of 9 to 14 years, who referred to Mashhad School of Dentistry. If there is a strong association between the dental developmental stages and skeletal growth spurt, the need for providing hand radiography is eliminated and patient’s radiation dose is reduced.

## Material and Methods 

In the current study, fifty two patients aged 9-14 years, who referred to Orthodontic Department of Mashhad Dental School, were evaluated. The mentioned age range was chosen since pubertal growth spurt usually occurs within these years, which is clinically important (10). Panoramic and lateral cephalometric radiographs were performed on all the patients, before the start of treatment.

**Figure 1:**
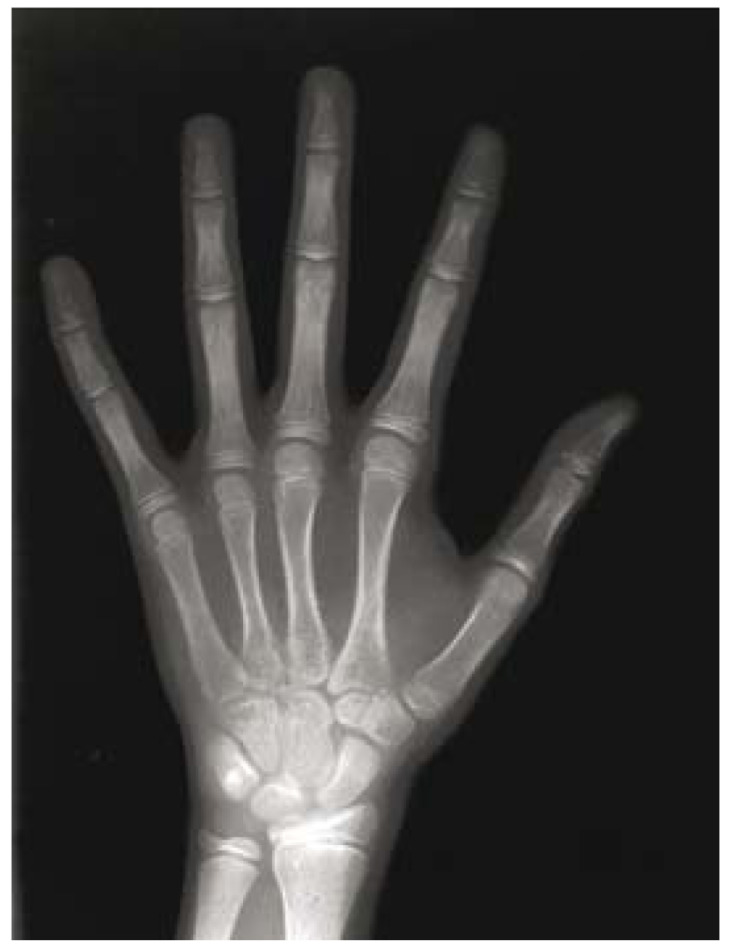
A posteroanterior hand-wrist radiograph from a 10-year-old girl. Equal width of epiphysis and diaphysis of the fifth finger middle phalanx resulted a SMI of 3.

After obtaining the informed consent, posteroanterior [PA] hand-wrist radiograph of the left hand [hand palm toward the cassette] with the fingers [slightly open] was performed. The exposure factors [kVp=60, mA=8, t = 0.2 s] were defined and the Planmeca 2002 CC system was utilized; also calcification of adductor sesamoid bone of the thumb was examined (Fig. 1). According to  Table 1, the rank of Skeletal Maturity Indicators [SMIs]was determined by Fishman’s method (4) for each patient.

**Table 1:**
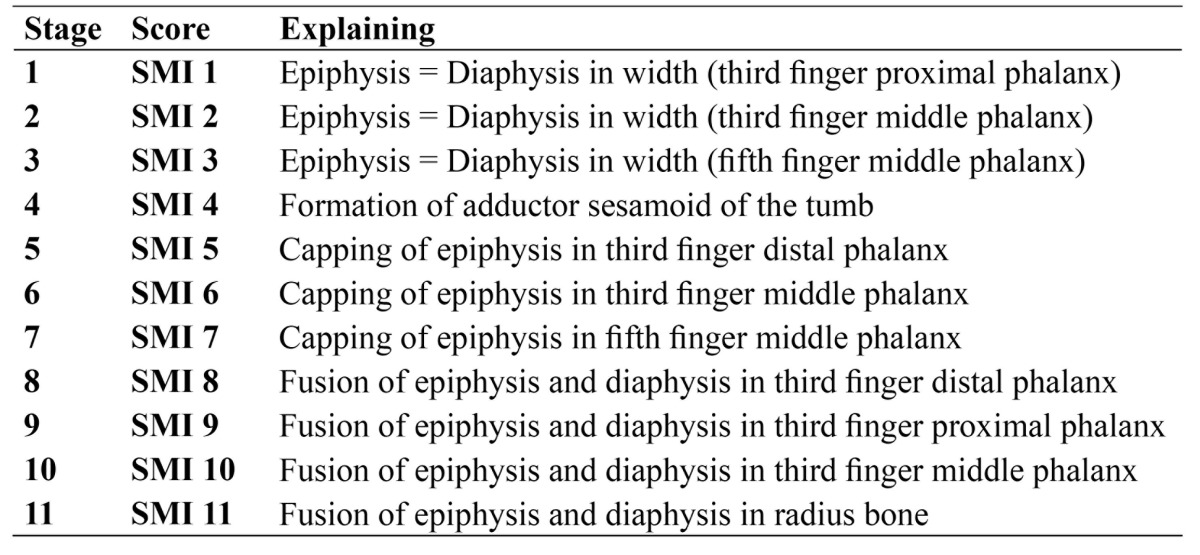
Skeletal maturity indicators (SMIs) criteria seen on a hand-wrist radiograph (4).

In panoramic radiography of the patients, dental developmental stages of mandibular second molars and canines teeth were separately determined by the Demirjian method (11). Each panoramic and PA hand-wrist radiographs were coded with a numerical ID so as to prevent observer bias, and the observer [A.B.], therefore, was not aware of the age or sex of the patients. After the passage of 2 months a random number of 50% [13 panoramic and 13 PA hand-wrist] of radiographs were re-examined by the same observer as a means of computing intra-observer reliability.

Statistical analyses were done using PASW® version 18. Independent t-test and Spearman correlation coefficient were used for data analysis. Intra-observer agreement was assessed using Kappa statistics. A *P*-value <0.05 was set to be statistically significant.

## Results

In this study, 52 patients aged 9-14 years were studied, among which 26 cases were females and 26 were males [the mean age of 10.62 ± 1.12 and 11.05 ± 1.05 years, respectively]. In order to increase the study accuracy, the patients’ age was calculated as decimal numbers; for instance, the age of 12 years and 3 months was recorded as 12.25 years ( Table 2).

**Table 2:**
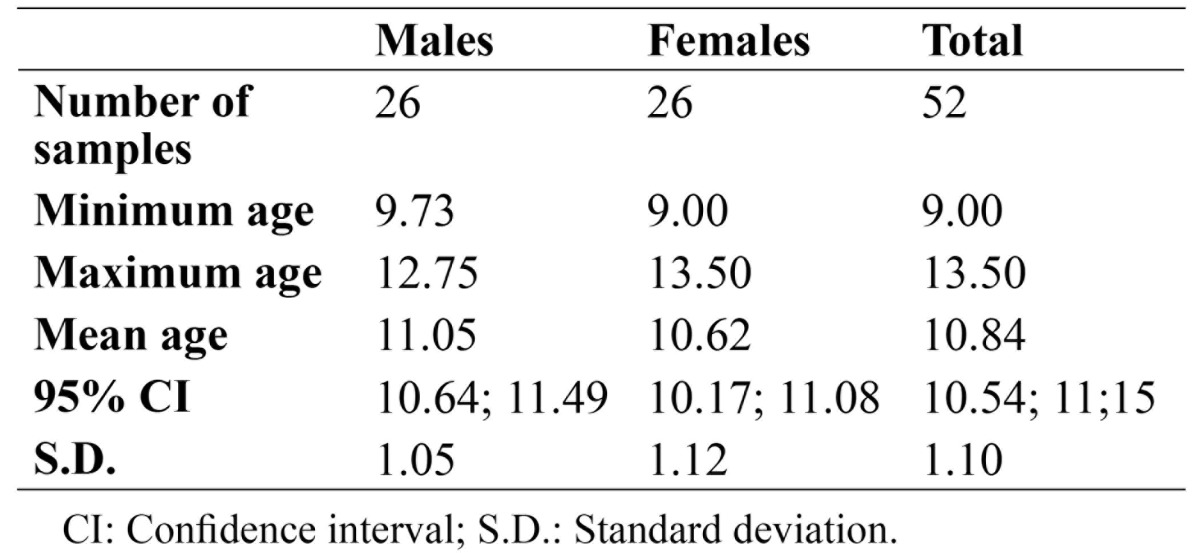
Descriptive findings of the studied samples.

Method reliability was verified using kappa statistics. For intra-observer agreement, the kappa values were 0.881 [0.816-0.946], and 0.901 [0.848-0.954] for panoramic and PA hand-wrist radiographs, respectively. According to  Table 1, and independent t-test, the mean age was not significantly different between the males and females [*p*=0.156]. According to the Fishman criteria for determining SMI, the patients were categorized based on their gender and SMI indices. The patients were divided into two groups of males and females. Results showed that correlation coefficients between SMI Index and developmental stages of the left and right canines and second molars were significant in the male group [*p*<0.05]. However, these correlation coefficients were not significant for the females ( Table 3).

**Table 3:**
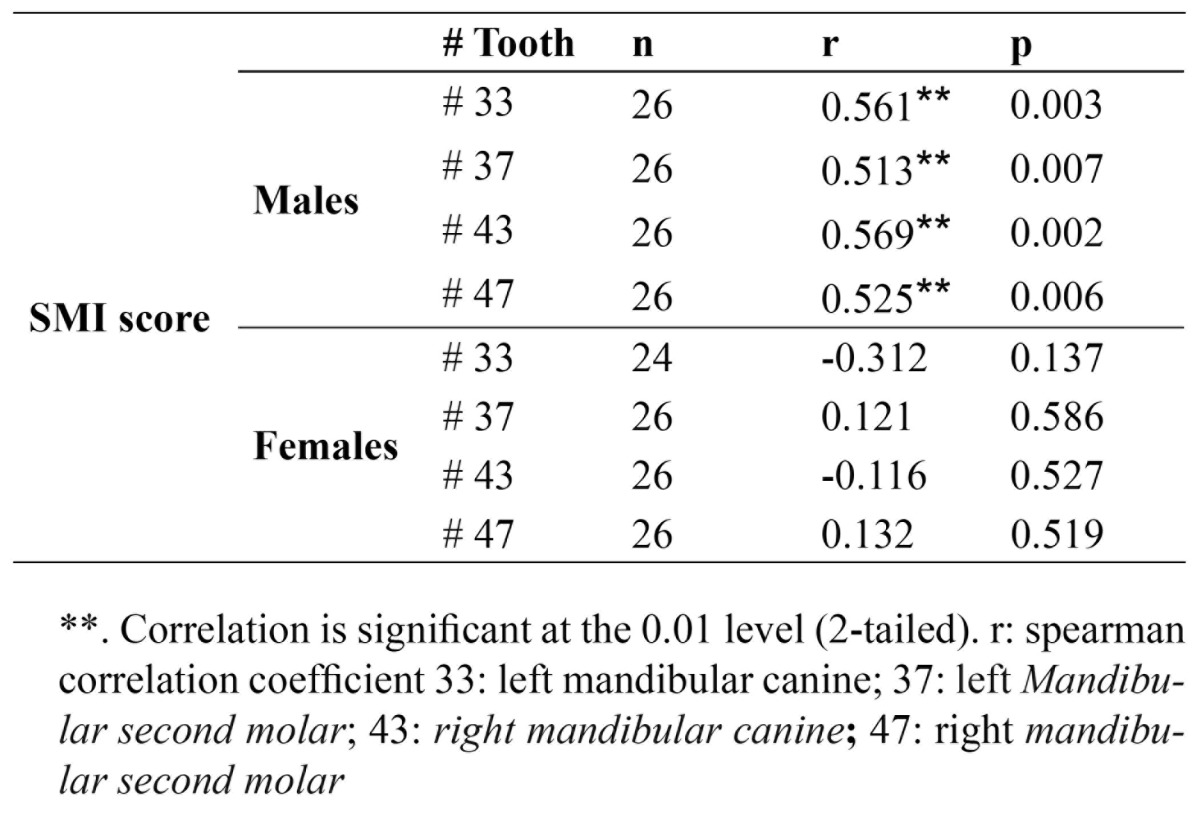
Spearman correlation coefficient between SMI and dental developmental tages of the studied teeth in both genders.

Another indicator which plays an important role in pubertal growth spurt, and indicates the initial stages or the start of pubertal growth spurt is the ossification of the thumb adductor sesamoid bones.

As the ranking nature of the formation of sesamoid bone and different stages of mandibular left and right canines and second molars teeth, the spearman correlation coefficients were calculated. Results showed that in the male group, there was a significant correlation between the development of sesamoid bones and developmental stages of the left mandibular canine [r=0.0520 and *p*=0.006] and the right counterpart [r=0.475 and *p*=0.014] ( Table 4).

**Table 4:**
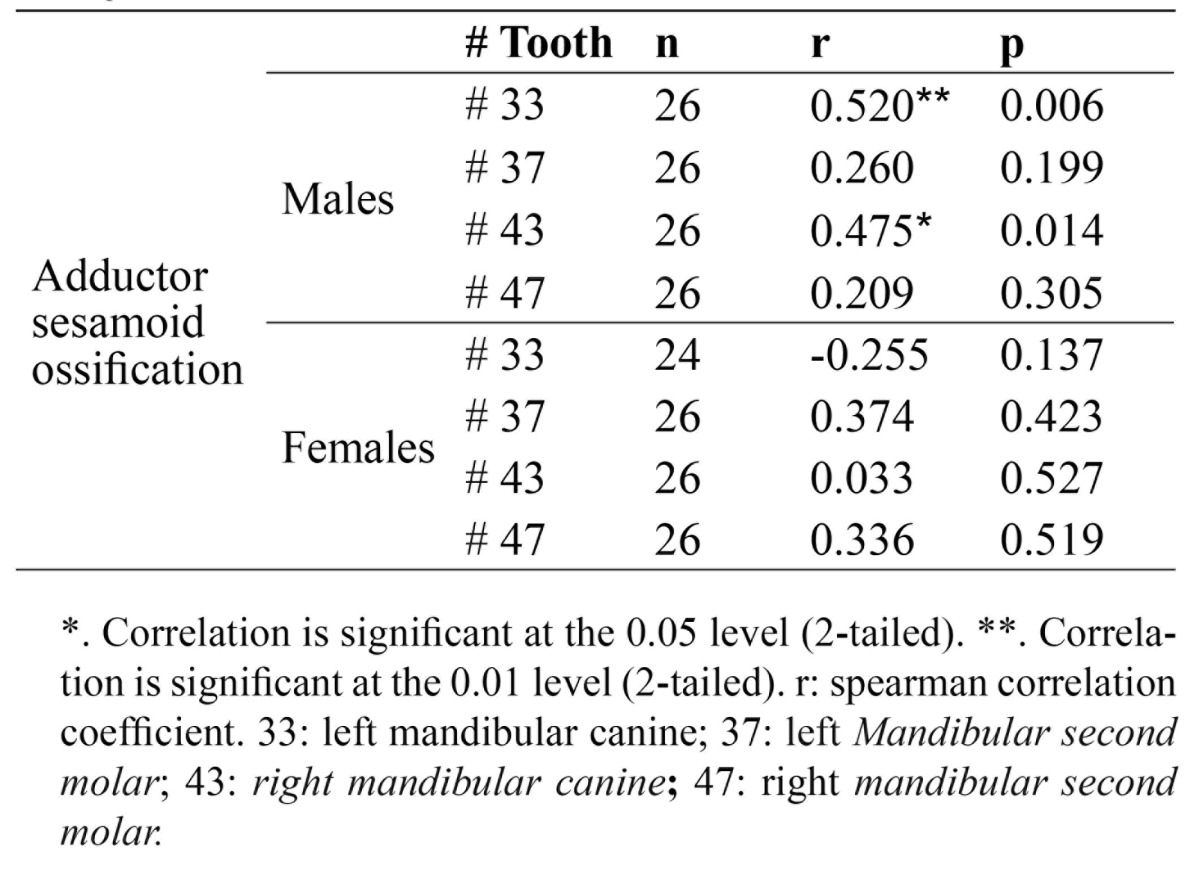
Spearman correlation coefficient between adductor sesamoid ossification and dental developmental stages of the studied teeth in both genders.

## Discussion

Assessment of the patients’ puberty and developmental events is one of the most basic and important elements in orthodontic treatment. Information regarding the pubertal growth spurt of the patients plays a significant role in the diagnosis, objectives, and selection of the treatment method (12). Clinical decisions about orthopedic treatments [with or without tooth extraction], use of extra-oral tractions, functional appliances, and maxillofacial surgeries are made based on the patient’s growth assessment. During the growing process, each bone undergoes a series of changes which are visible by radiography. Since each person has his/her own unique biological clock, the time of these changes is different in each individual; therefore, it is important to evaluate the growth status of each person (13).

One of the conventional methods to evaluate skeletal development is the use of hand-wrist radiography (12). Currently, Fishman skeletal pubertal system is commonly used for this purpose (14).

Previous studies showed that dental puberty especially that of the mandibular canines teeth is associated with the developmental stages of skeletal maturity (8,9,12,15,16). If there is a strong correlation between the stages of dental development and skeletal maturation, dental development can be used as the first diagnostic criteria for evaluating pubertal growth spurt. The simple diagnosis of dental developmental stages along with the accessibility to intra-oral or panoramic radiography for most orthodontic patients are the reasons which encourage us to use the dental developmental stages as an alternative to hand-wrist radiographs.

Previous studies reported that racial differences affect this association (12). Unfortunately, no study in Iran has been conducted in this field, or if performed, no information is at hand. This study was performed with the aim to evaluate the association between dental developmental stages and phases of skeletal maturity.

The findings of this study indicated that the correlation coefficients between SMI Index and the left and right canines were significant in males [r=0.561, *p*=0.003 and r=0.569, *p*=0.002, respectively]; however, they were not significant in the female group. A strong correlation was found between the calcification of mandibular canine and skeletal maturation in the studies of Krailassiri *et al*. (12), Chertkow (8), Chertkow and Fatti (17), Siera (18), and Coutinho and colleagues (9). In the present study, despite the smaller sample size in comparison with the aforementioned studies, the relationship between skeletal maturation and developmental stages of mandibular canine was significant, with relatively high correlation coefficient.

The different results regarding the male and female groups in our study indicate a two-fold pattern in terms of gender. Unlike our study, Chertkow and Fatti (17) in their study reported that no significant difference in the canine tooth was found between the two genders in terms of the distribution of tooth mineralization stages. On the other hand, the studies of Chertkow (8), Krailassiri *et al*. (12), and Uysal *et al*. (16) showed that tooth mineralization associated with skeletal maturation occurs in an earlier period in boys.

Krailassiri and colleagues in a study in Thailand showed a significant association between the stages of dental calcification and the phases of skeletal maturity [r=0.31-0.69, *p* <0.01] (12). Among males, the most significant correlation was related to mandibular second premolar, first premolar, second molar and canine teeth, respectively. However, the most significant association in females was related to second premolar, second molar, first premolar and canine teeth, respectively. Uysal and colleagues’s study in Turkey showed the correlation between dental development and skeletal maturation in males was within the range of 0.414 to 0.706, and in females in the range of 0.490 to 0.826 [*p*<0.01] (16). For both males and females, the highest correlation was observed in the second molar and the lowest in the third molar teeth. The findings of the present study indicated that the correlation between the canines and second molars development and skeletal maturation is remarkable in male subjects. Our study differed from the mentioned studies, since no statistically significant relationship was found between the developmental stages of the studied teeth and hand skeletal maturation, in females. This may be due to the differences in the sample size which determine the strength of the study; also racial differences are highly important. Variations in climatic factors and the nutritional and socioeconomic level can be considered as the source of racial differences.

Another indicator which plays an important role in pubertal growth spurt and indicates the initial stages or start of pubertal growth spurt is the ossification of thumb adductor sesamoid bones; the fourth step in the classification of Fishman [SMI 4] represents the emergence of thumb adductor sesamoid (4).

The study of Chertkow (8), and Chertkow and Fatti (17) reported that based on Demirjian category, the G phase of the developmental stages of mandibular canine is associated with the emergence of sesamoid in 77% of cases. In the study of So (19), there was no close relationship between sesamoid ossification and canine development.

The findings of our study indicated that the significant correlation between the development of sesamoid bones and developmental stages of mandibular left [r=0.0520 and *p*=0.006] and the right [r=0.475 and *p*=0.014] canines was observed only in males. However, our findings are not reliable enough to be compared with other studies, since the sample size of the current study is smaller, and this shows that further studies with a larger sample size are required.

## Conclusions

Findings of this study showed that the correlation between dental developmental stages and skeletal maturity were significant in males only; thus, different skeletal maturity patterns in males and females could be perceptible.
